# Systematic genome editing of the genes on zebrafish Chromosome 1 by CRISPR/Cas9

**DOI:** 10.1101/gr.248559.119

**Published:** 2020-01

**Authors:** Yonghua Sun, Bo Zhang, Lingfei Luo, De-Li Shi, Han Wang, Zongbin Cui, Honghui Huang, Ying Cao, Xiaodong Shu, Wenqing Zhang, Jianfeng Zhou, Yun Li, Jiulin Du, Qingshun Zhao, Jun Chen, Hanbing Zhong, Tao P. Zhong, Li Li, Jing-Wei Xiong, Jinrong Peng, Wuhan Xiao, Jian Zhang, Jihua Yao, Zhan Yin, Xianming Mo, Gang Peng, Jun Zhu, Yan Chen, Yong Zhou, Dong Liu, Weijun Pan, Yiyue Zhang, Hua Ruan, Feng Liu, Zuoyan Zhu, Anming Meng, The ZAKOC Consortium

**Affiliations:** 1 State Key Laboratory of Freshwater Ecology and Biotechnology, Institute of Hydrobiology, Innovation Academy for Seed Design, Chinese Academy of Sciences, Wuhan, Hubei, 430072, China;; 2 Key Laboratory of Cell Proliferation and Differentiation of the Ministry of Education, Peking University Genome Editing Research Center, College of Life Sciences, Peking University, Beijing, 100871, China;; 3 School of Life Sciences, Southwest University, Chongqing, 400715, China;; 4 Guangdong Medical University, Zhanjiang, Guangdong, 524023, China;; 5 Center for Circadian Clocks, Soochow University, Suzhou, Jiangsu, 215123, China;; 6 School of Life Sciences and Technology, Tongji University, Shanghai, 200092, China;; 7 Guangzhou Institutes of Biomedicine and Health, Chinese Academy of Sciences, Guangzhou, Guangdong, 510530, China;; 8 Division of Cell, Developmental and Integrative Biology, School of Medicine, South China University of Technology, Guangzhou, Guangdong, 510006, China;; 9 School of Medicine and Pharmacy, Ocean University of China, Qingdao, Shandong, 266100, China;; 10 State Key Laboratory of Neuroscience, Institute of Neuroscience, Chinese Academy of Sciences, Shanghai, 200031, China;; 11 Model Animal Research Center, Nanjing University, Nanjing, Jiangsu, 210093, China;; 12 College of Life Sciences, Zhejiang University, Hangzhou, Zhejiang, 310058, China;; 13 Department of Biology, South University of Science and Technology of China, Shenzhen, Guangdong, 518055, China;; 14 Institute of Biomedical Sciences, East China Normal University, Shanghai, 200062, China;; 15 College of Life Sciences, Institute of Molecular Medicine, Peking University, Beijing, 100871, China;; 16 School of Life Sciences, Yunnan University, Kunming, Yunnan, 650091, China;; 17 School of Life Sciences, Fudan University, Shanghai, 200433, China;; 18 State Key Laboratory of Biotherapy, West China Hospital, Sichuan University, Chengdu, Sichuan, 610041, China;; 19 Institutes of Brain Science, Fudan University, Shanghai, 200433, China;; 20 Sino-French Research Center for Life Sciences and Genomics, Rui-Jin Hospital, Shanghai Jiao Tong University School of Medicine, Shanghai, 200025, China;; 21 Institute of Health Sciences, Chinese Academy of Sciences & Shanghai Jiao Tong University School of Medicine, Shanghai, 200025, China;; 22 CAS Key Laboratory of Nutrition, Metabolism and Food Safety, Shanghai Institute of Nutrition and Health, Chinese Academy of Sciences, Shanghai, 200031, China;; 23 State Key Laboratory of Membrane Biology, Institute of Zoology, Chinese Academy of Sciences, Beijing, 100101, China;; 24 School of Life Sciences, Tsinghua University, Beijing, 100084, China;; 26 State Key Laboratory of Freshwater Ecology and Biotechnology, Institute of Hydrobiology, Innovation Academy of Seed Design, Chinese Academy of Sciences, Wuhan, Hubei, 430072, China;; 27 Key Laboratory of Cell Proliferation and Differentiation of the Ministry of Education, Peking University Genome Editing Research Center, College of Life Sciences, Peking University, Beijing, 100871, China;; 28 School of Life Sciences, Southwest University, Chongqing, 400715, China;; 29 School of Life Sciences, Shandong University, Qingdao, 266237, China;; 30 Center for Circadian Clocks, Soochow University, Suzhou, Jiangsu, 215123, China;; 31 School of Life Sciences and Technology, Tongji University, Shanghai, 200092, China;; 32 Guangzhou Institutes of Biomedicine and Health, Chinese Academy of Sciences, Guangzhou, Guangdong, 510530, China;; 33 Division of Cell, Developmental and Integrative Biology, School of Medicine, South China University of Technology, Guangzhou, Guangdong, 510006, China;; 34 School of Medicine and Pharmacy, Ocean University of China, Qingdao, Shandong, 266100, China;; 35 State Key Laboratory of Neuroscience, Institute of Neuroscience, Chinese Academy of Sciences, Shanghai, 200031, China;; 36 Model Animal Research Center, Nanjing University, Nanjing, Jiangsu, 210093, China;; 37 College of Life Sciences, Zhejiang University, Hangzhou, Zhejiang, 310058, China;; 38 Department of Biology, South University of Science and Technology of China, Shenzhen, Guangdong, 518055, China;; 39 Institute of Chinese Medical Sciences, University of Macau, Macau, China;; 40 Institute of Biomedical Sciences, Shanghai Key Laboratory of Regulatory Biology, East China Normal University, Shanghai, 200062, China;; 41 College of Life Sciences, Institute of Molecular Medicine, Peking University, Beijing, 100871, China;; 42 College of Animal Sciences, Zhejiang University, Hangzhou, Zhejiang, 310058, China;; 43 State Key Laboratory for Conservation and Utilization of Bio-Resources, Kunming, 650091, China;; 44 Center for Life Sciences, School of Life Sciences, Yunnan University, Kunming, 650091, China;; 45 State Key Laboratory of Molecular Developmental Biology, Institute of Genetics and Developmental Biology, Chinese Academy of Sciences, Beijing, 100101, China;; 46 School of Life Sciences, Fudan University, Shanghai, 200433, China;; 47 State Key Laboratory of Biotherapy, West China Hospital, Sichuan University, Chengdu, Sichuan, 610041, China;; 48 Institutes of Brain Science, Fudan University, Shanghai, 200433, China;; 49 Sino-French Research Center for Life Sciences and Genomics, Rui-Jin Hospital, Shanghai Jiao Tong University School of Medicine, Shanghai, 200025, China;; 50 Institute of Health Sciences, Chinese Academy of Sciences & Shanghai Jiao Tong University School of Medicine, Shanghai, 200025, China;; 51 CAS Key Laboratory of Nutrition, Metabolism and Food Safety, Shanghai Institute of Nutrition and Health, Chinese Academy of Sciences, Shanghai, 200031, China;; 52 State Key Laboratory of Membrane Biology, Institute of Zoology, Chinese Academy of Sciences, Beijing, 100101, China;; 53 School of Life Sciences, Tsinghua University, Beijing, 100084, China;; 54 Present address: Affiliated Hospital of Guangdong Medical University, Zhanjiang, 524001, China

## Abstract

Genome editing by the well-established CRISPR/Cas9 technology has greatly facilitated our understanding of many biological processes. However, a complete whole-genome knockout for any species or model organism has rarely been achieved. Here, we performed a systematic knockout of all the genes (1333) on Chromosome 1 in zebrafish, successfully mutated 1029 genes, and generated 1039 germline-transmissible alleles corresponding to 636 genes. Meanwhile, by high-throughput bioinformatics analysis, we found that sequence features play pivotal roles in effective gRNA targeting at specific genes of interest, while the success rate of gene targeting positively correlates with GC content of the target sites. Moreover, we found that nearly one-fourth of all mutants are related to human diseases, and several representative CRISPR/Cas9-generated mutants are described here. Furthermore, we tried to identify the underlying mechanisms leading to distinct phenotypes between genetic mutants and antisense morpholino-mediated knockdown embryos. Altogether, this work has generated the first chromosome-wide collection of zebrafish genetic mutants by the CRISPR/Cas9 technology, which will serve as a valuable resource for the community, and our bioinformatics analysis also provides some useful guidance to design gene-specific gRNAs for successful gene editing.

Zebrafish (*Danio rerio*) has been extensively used as a vertebrate model organism in developmental biology and genetic studies, partly due to the transparency of the early embryos and feasibility of obtaining a large quantity of progeny. In the past decades, large-scale investigation of gene function in development and genetics has been achieved primarily through N-ethyl-N-nitrosourea (ENU)-mediated mutagenesis (Solnica-Krezel et al. 1994) and retroviral mutagenesis (Amsterdam et al. 1999; Golling et al. 2002), also referred to as ‘forward’ genetics (from phenotypes to genes) (Huang et al. 2012). Compared to a ‘forward’ genetic study, ‘reverse’ genetics (from genes to phenotypes) can precisely mutate the target sites of interest in the genome and systematically identify the mutated phenotypes (Wienholds et al. 2003; Kettleborough et al. 2013; Varshney et al. 2013). Currently, with the development and application of engineered endonucleases, including zinc finger nucleases (ZFN) (Doyon et al. 2008), transcription activator-like effector nucleases (TALEN) (Moore et al. 2012), and the CRISPR/Cas system (such as the CRISPR/Cas9 system) (Li et al. 2016; Varshney et al. 2016), the ‘reverse’ genetic study has evolved rapidly. The flexibility of the CRISPR/Cas9 system and data from the completed zebrafish genome sequencing project make it possible to systematically knock out every single gene in zebrafish (Howe et al. 2013; Hwang et al. 2013; Varshney et al. 2015). However, properties of gene targeting via the CRISPR/Cas9, such as the sequence bias of targetable or highly efficient sites, remain elusive and need to be substantially characterized.

To generate a comprehensive genetic resource to facilitate zebrafish research for the community, we aim to knock out all the annotated genes in zebrafish Chromosome 1, a project involving 38 laboratories from 24 institutions in China. To our knowledge, this is the first report on systematic genome targeting spanning an entire chromosome of an organism in vertebrates with the use of the CRISPR/Cas9 technology.

## Results

### Selection of target genes on zebrafish Chromosome 1

The ∼1.5-giga base-pair (Gbp) haploid nuclear genome of zebrafish comprises 25 chromosomes (Howe et al. 2013). It is estimated that there are ∼32,000 genes in the genome, including ∼26,000 coding genes and ∼6000 noncoding genes, and roughly 1300 genes per chromosome on average. The size of Chromosome 1 of zebrafish was reported to be about 60 mega base-pairs (Mbp), constituting ∼4% of the whole genome and containing 1418 genes (according to Zv9 release 60, 2013-01) (Table 1). Except for seven pseudogenes and 78 rRNA genes present on Chromosome 1, a total of 1333 genes were considered for the gene targeting attempts, consisting of 1202 coding genes and 131 noncoding genes (including 31 microRNAs and six long intergenic noncoding RNAs) (Table 1). For convenience, all of the selected target genes were numbered according to their order along Chromosome 1, with the prefix “zko.” A full list of the zko genes can be found in the Supplemental Data (Supplemental Table S1).

**Table 1.**
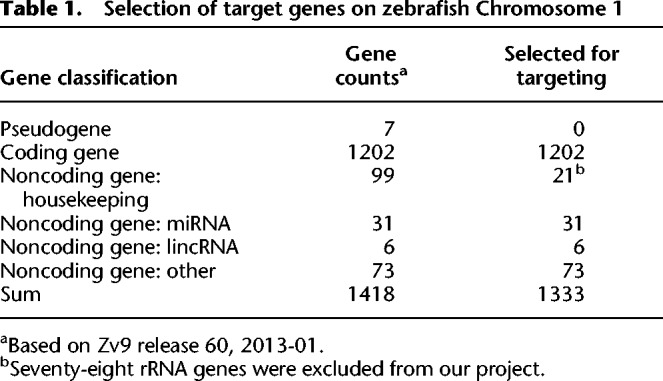
Selection of target genes on zebrafish Chromosome 1

Among these 1333 target genes (zko genes), only 109 genes had been studied with morpholino (MO) antisense oligonucleotides, 690 genes were recorded to have mutated/modified alleles in the zebrafish information network (ZFIN), an open-access online database for zebrafish research, and mutations for 606 genes had been identified from the Sanger Institute Zebrafish Mutation Project (Supplemental Table S1), while the majority of the target genes had not been subjected to mutational analysis. Furthermore, most of the mutated genes are coding genes, whereas only 39 noncoding genes had available mutations, most of which are point mutations generated through ENU mutagenesis.

### Summary of targeted genes/mutations on zebrafish Chromosome 1

In order to disrupt the target genes completely, we adopted the strategy of using a single gRNA to generate indel mutations for the coding genes and a pair of gRNAs to produce genomic deletions for the noncoding genes (Supplemental Fig. S1). As to some coding genes, several gRNAs were designed and validated at the same time. Therefore, at least one gRNA for each coding gene and one pair of gRNAs for each noncoding gene was designed and tested. In rare cases, more than 10 gRNAs were designed and tested for one coding gene, such as *zko187* (*chtf18*), since none of the 10 gRNAs gave positive results.

Up until now, 1029 (77.2%) out of 1333 zko genes have been successfully mutated. Mutations were detected at least in founder embryos after injection of gRNA(s) together with Cas9 mRNA, including 962 coding genes and 67 noncoding genes. Mutations for 438 genes are first reported in this study. In total, 2277 gRNAs have been designed and tested, and 1086 (47.8%) among them showed to be efficient (Supplemental Table S1). After screening the inheritance of mutant alleles, we have successfully obtained 1039 germline mutant alleles in 636 genes (61.8% against 1029 genes), among which 703 alleles corresponding to 452 genes have given rise to the F_2_ generation (Table 2; Supplemental Table S2).

**Table 2.**
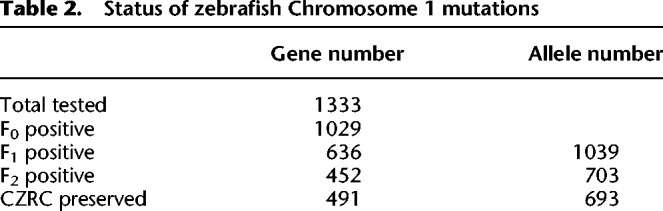
Status of zebrafish Chromosome 1 mutations

### Characterization of the features of the CRISPR/Cas9 target sequences

It is well known that the success rate of gene targeting via the CRISPR/Cas9 system is not 100%. Detectable mutations cannot be effectively induced at certain gRNA target sites, although no obvious rules could explain this phenomenon yet. To better understand the targeting capabilities of the CRISPR/Cas9 system in zebrafish, we analyzed the features of CRISPR/Cas9 target sequences, based on the large amounts of gRNAs tested in this study.

### GC-content distribution in tested target sites

**Figure 1.**
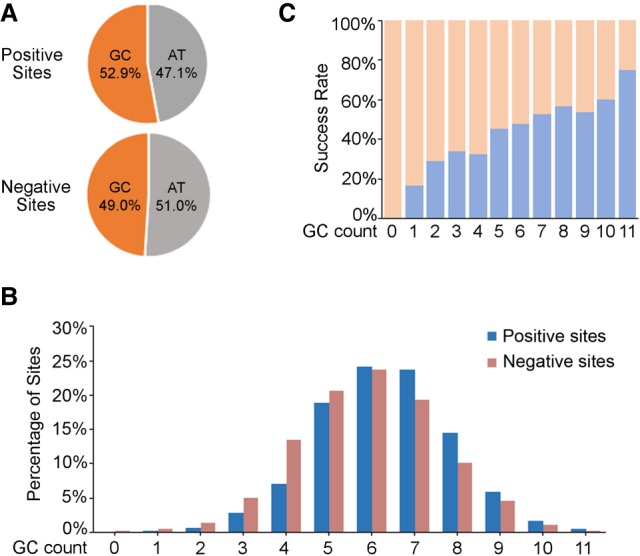
GC-content distribution in the 12-nt seed sequence of all the tested target sites (including 1086 positive sites and 1191 negative sites). (*A*) GC percentage of positive and negative target sites at the seed region, respectively. (*B*) GC percentage distribution of the seed region in all the tested target sites. GC count: number of G or C nucleotides in the 12-nucleotide (nt) seed sequence of the target sites. (*C*) Correlation of the positive rate of target sites with different GC percentages of the seed region. GC count: number of G or C nucleotides in the 12-nt seed sequence of the target sites.

All the tested gRNA target sites were collected for the analyses of target site features, including both “positive” ones (1086 in total that gave rise to mutations in F_0_ fish) and “negative” ones (1191 in total that did not result in mutations in founder embryos) (Supplemental Table S1). We first compared the GC content between the two types of target sequences. The average GC content of all the tested target sites (the protospacer adjacent motif [PAM] sequence NGG was ignored, i.e., only protospacer sequences were used for the calculation) is 53.0%. The slightly high GC content could be partially explained by the presence of one or two Gs at the beginning of the target sequence, which is required for an efficient in vitro transcription of gRNAs by the T7 RNA polymerase. When only considering the more important 12-nucleotide (nt) seed sequence, the average GC content for all the target sites is reduced to 50.9%, close to expectation for unbiased design of target sequences. The GC content at the seed region is significantly higher in the 1086 positive target sites than in the 1191 negative ones, with 52.9%, on average, for the positive sites and 49.0%, on average, for the negative ones (Fig. 1A). This suggests that higher GC content in the seed region of the target site is more likely to result in successful mutation. This tendency can be seen more clearly when comparing the distribution of GC content at the seed region in the positive sites, where 70.4% (765/1086) of sites show GC content >50%, and that in the negative sites, where only 58.9% (703/1191) of sites show GC content >50% (Fig. 1B). In addition, this is revealed by the strong positive correlation of the success rate of targeting with the GC content of the seed sequences (Fig. 1B,C).

### Distribution of nucleotide motifs in tested target sites

**Figure 2.**
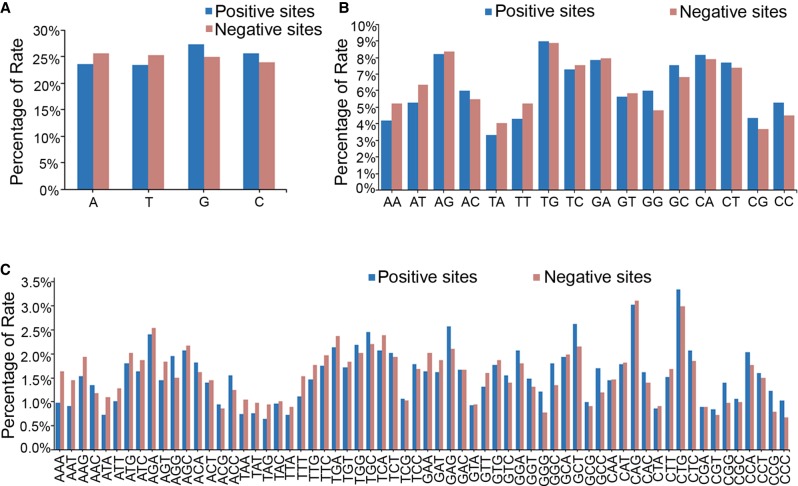
Distribution of nucleotide motifs in the 12-nt seed region of all the tested target sites. (*A*) The statistical data showing the distribution of each single nucleotide in the seed region of the target sites. (*B*) The statistical data showing the distribution of 2-nt motifs in the seed region of the target sites. (*C*) The statistical data showing the distribution of 3-nt motifs in the seed region of the target sites.

Positions of nucleotides and nucleotide context might influence the targeting efficiency of the CRISPR/Cas9 system. We next assessed the base composition as well as the distribution of single nucleotides and nucleotide motifs in all the tested target sites. No significant difference was detected regarding single nucleotide distribution or ratio in the first 18 nt adjacent to the PAM region between the positive sequences and the negative ones, though there are variations among different nucleotides and at different positions (Fig. 2A; Supplemental Fig. S2), and the enrichment for G and C nucleotides in the positive sites is evident almost at every position (Supplemental Fig. S3). One of the few exceptions for this phenomenon is the composition of nucleotide C at position −1, the first nucleotide immediately upstream of the PAM, where negative sites are dominant for this nucleotide, suggesting that C at this position might have a negative impact on targeting efficiency (Supplemental Fig. S3D). The significantly high percentage of nucleotide G at the last two positions (−19 and −20 relative to the PAM) is due to the obligate requirement for the presence of two terminal Gs for efficient in vitro transcription of gRNAs by T7 RNA polymerase. The distribution of PAM sequences also did not show a significant difference between the positive and negative target sites, although GGG and CGG appear slightly more frequently in the positive sites than the negative ones (Supplemental Fig. S2A).

We further compared the distribution of 2-nt and 3-nt motifs in the tested target sites and their potential relationship with mutation rate. Among all the 16 different combinations of 2-nt motifs and 64 different 3-nt motifs, we found that target sites containing motifs such as “C/GC/G” dinucleotides or “C/GNC/G” trinucleotides are more likely to induce mutations (Fig. 2B,C). This observation is consistent with our GC content analysis mentioned above as well as a previous report showing that the activity of the CRISPR/Cas9 system is GC content–dependent (Labuhn et al. 2018).

### Collection and distribution of the zko alleles

The F_2_ fish lines resulting from the ZKO project were collected and their identities were confirmed by the China Zebrafish Resource Center (CZRC) (http://en.zfish.cn) through Sanger sequencing (Supplemental Data File S1). Sperm samples were obtained from the genotype-verified fish lines and frozen for cryopreservation. In 954 confirmed mutations (Supplemental Table S2), there are 24 big indels (>200 bp) with two gRNA targeting; thus, the rest of the 930 mutant alleles were analyzed for indel characteristics. The results showed that 61.4% (571/930) are deletions with an average size of 10.9 bp, 11.1% (103/930) are insertions with an average size of 6.4 bp, and 27.5% (256/930) are indels with an average insertion of 10.3 bp and deletion of 9 bp. In those 930 mutant alleles, 11.6% (108/930) are in-frame mutations and 88.4% (822/930) are frame-shift mutations.

A total of 693 alleles were successfully preserved by CZRC (Supplemental Table S3). All the nonsense zko alleles are listed at the websites of ZFIN (http://zfin.org/action/publication/ZDB-PUB-171002-4/feature-list) or CZRC (http://www.zfish.cn/TargetList.aspx). On the information page of each zko gene, the gene name, Ensembl ID, gRNA target site, target position, allele name, allele sequence, and a short description of the predicted protein product are shown. In addition, the detailed genome sequence flanking the target site, as well as a cluster analysis of the pairwise alignments (CLUSTAL) of the allele sequence and the wild-type genome sequence are presented as Portable Document Format (PDF) files. All the zko alleles could be ordered from CZRC through an online ordering system (http://en.zfish.cn/inforscanEN/173.html).

### Characterization of development- and disease-related phenotypes from the zko mutants

**Figure 3.**
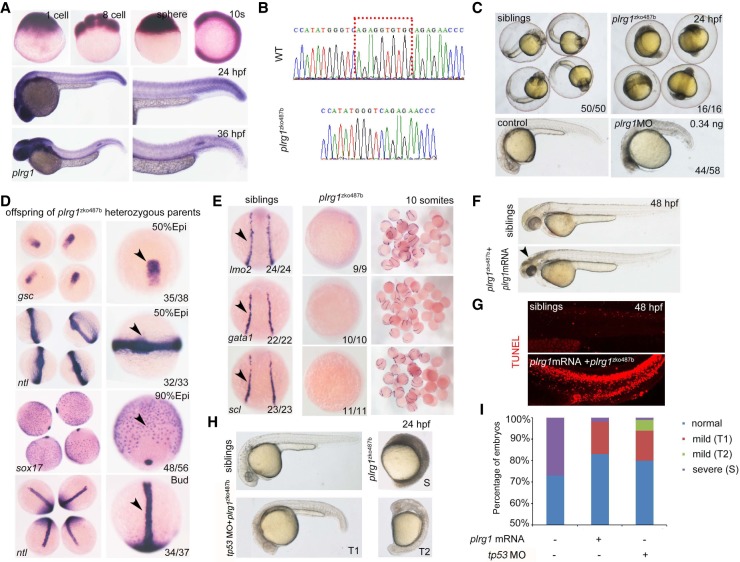
Characterization of *plrg1* mutant generated by the CRISPR/Cas9 system. (*A*) Whole-mount in situ hybridization (WISH) showing the expression of *plrg1* at different developmental time points from the one-cell stage to 36 h postfertilization (hpf). (*B*) The comparison of genomic DNA sequences between wild type (WT) and *plrg1* mutants with 10-base pair deletion. (*C*) The *plrg1* mutants and morphants showed severe developmental defects, with black head and small body compared to the siblings and control embryos, respectively, at 24 hpf. (*D*) WISH showing the expression of *gsc* and *ntl* at the 50% epiboly stage, *sox17* at 90% epiboly stage, and *ntl* at bud stage in the offspring of *plrg1* heterozygous parents. The *right* panels show the magnified images, and the black arrowheads indicate corresponding expression of *gsc* at the dorsal margin, *ntl* at anterior axial hypoblast, forerunner cell group, and margin, and *sox17* at endoderm and forerunner cells. (*E*) WISH showing the expression of *lmo2*, *gata1*, and *scl* at lateral plate mesoderm in the siblings and *plrg1* mutants. (*F*) Overexpression of the *plrg1* full-length mRNA can rescue *plrg1* mutants until 4 dpf. The body defects of mutants were rescued efficiently by mRNA overexpression, but there is still a black head at 48 hpf (arrowhead) in *plrg1* mutants. (*G*) TUNEL assay displays that there are obvious apoptotic signals at 48 hpf in *plrg1* mutants injected with *plrg1* mRNA. (*H*) Injection of *tp53* morpholino can rescue the developmentally defective phenotype of *plrg1* mutants efficiently at 24 hpf. There are three subtypes of defective embryos, and we describe the siblings as normal, *plrg1* mutants as severe (S), and partial rescued mutants as mild (T1) and mild (T2). (*I*) The quantification of *plrg1* mutant embryos in different treatment groups shown in *H*.

We then characterized the phenotypes in the verified mutants generated using the CRISPR/Cas9 system. We exemplified the typical paradigm for detailed phenotype characterization using pleiotropic regulator 1 that is encoded by *plrg1*^*zko487*^. *plrg1* is maternally expressed and enriched in the head and tail region at 24 h postfertilization (hpf) and 36 hpf as shown by whole-mount in situ hybridization (WISH) (Fig. 3A). The homozygous *plrg1* mutants with a 10-bp deletion (Fig. 3B) displayed significant developmental defects, which can be distinguished readily based on the features of black head and smaller body at 24 hpf (Fig. 3C). To verify whether the phenotype of *plrg1* is gene-specific, we designed antisense morpholinos to knock down *plrg1* gene expression. The observed embryonic defects in *plrg1* morphants were similar to those found in homozygous *plrg1* mutants (Fig. 3C). We examined the expression of early development marker genes including *gsc*, *ntl*, and *sox17* and were unable to detect obvious defects in the homozygous *plrg1* mutants until the bud stage (Fig. 3D). In contrast, the expression of hematopoietic development-related genes *lmo2*, *gata1*, and *scl* was decreased along with abnormal development in the *plrg1* mutants (Fig. 3E). To further exclude the off-targeting effects of the CRISPR/Cas9 system, we synthesized the full-length mRNA of *plrg1* to see whether we can rescue the *plrg1* mutants. Our results showed that the *plrg1* mRNA could rescue the early defects in the homozygous mutants, leading to their survival up to 4 d postfertilization (dpf), despite the apoptosis noticed (Fig. 3F,G).

Previous studies have demonstrated that Plrg1 controls cell growth by negatively regulating Tp53 class mediators (Kleinridders et al. 2009; Sorrells et al. 2012). We thus applied the *tp53* MO to see whether the defects of *plrg1* mutants can be alleviated. Our results showed that the mutants were efficiently rescued by injection of the *tp53* MO (Fig. 3H). Quantification of the embryos in different groups confirmed that the *plrg1* mutants were rescued efficiently both by *plrg1* mRNA and by the *tp53* MO (Fig. 3I). Moreover, genes that are essential for early zebrafish development were identified by analyzing corresponding homozygous mutants, such as two homozygous alleles of *tolloid-like 1* (*tll1*), *tll1*^*zko395a*^, *and tll*^*zko395b*^, and one homozygous allele of *pi4k2a*^*zko1099a*^ (Supplemental Fig. S4).

Among the identified mutants by the CRISPR/Cas9 system, we found that nearly one in four of the mutated genes in our study were associated with human diseases (Supplemental Table S1). For example, RUNX family transcription factor 1 (Runx1), which is encoded by *runx1*^*zko52*^, is essential for hematopoietic stem/progenitor cell (HSPC) production in the aorta-gonad-mesonephros (AGM) region during embryonic hematopoiesis. Extensive studies showed that abnormal *runx1* expression correlates with acute myeloid leukemia and platelet disorder (Rao 2013; Sood et al. 2017). We examined the hematopoietic phenotypes in the *runx1* mutants and found that *c-myb* (HSPC marker gene) expression in AGM at 36 hpf was decreased in these mutants compared to the control embryos (Supplemental Fig. S4A), which is consistent with previously published data (Sood et al. 2010). In another example, zebrafish with *gyg2*^*zko624a*^ mutation displayed increased blood glucose content (Supplemental Fig. S4B). Similarly, *GYG2* mutation has been characterized as a pathogenic mutation in human Leigh syndrome, an early-onset progressive neurodegenerative disorder resulting from defective glycogen synthesis (Imagawa et al. 2014). Taken together, by following a conventional pipeline for phenotype identification of the generated mutants, we revealed a large number of previously uncharacterized phenotypes associated with development and disease.

### Comparison between genetic mutants generated by CRISPR/Cas9 and their corresponding morphants by antisense MO knockdown

**Figure 4.**
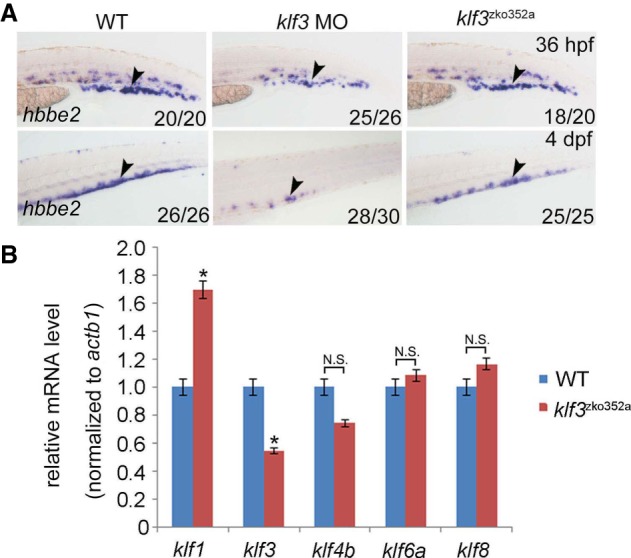
Phenotypic comparison between *klf3* mutants and morphants. (*A*) WISH showing the expression of *hbbe2* (also known as *βe2-globin*) in *klf3* WT, morphants, and mutants. The black arrowheads indicate *hbbe2* expression in erythroid lineages. (*B*) Quantitative real-time PCR showing the expression of Klf members *klf1*, *klf3*, *klf4b*, *klf6a*, and *klf8* in *klf3* mutants.

During the past two decades, reverse genetic approaches, including ZFNs, TALENs, and CRISPR/Cas9, have been developed rapidly and used extensively in genome editing and disease modeling (Boch et al. 2009; Urnov et al. 2010; Jinek et al. 2012; Wiedenheft et al. 2012; Hwang et al. 2013). However, a large proportion of mutants generated by reverse genetics methods failed to recapitulate published morpholino-induced phenotypes (morphants) in zebrafish (Kok et al. 2015). Moreover, it was reported that genetic compensation can be induced by deleterious mutations (El-Brolosy et al. 2019; Ma et al. 2019), for example, as observed in zebrafish *egfl7* mutants (Rossi et al. 2015). We also observed phenotypes inconsistent between newly generated mutants and their corresponding morphants. For instance, the mutation of *dpy30*^*zko989*^, encoding a histone methyltransferase complex subunit, showed no obvious phenotypes of hematopoietic cell differentiation (Supplemental Fig. S5A,B), which is inconsistent with the published morphant phenotypes that exhibit defective erythropoiesis and lymphopoiesis (Yang et al. 2014). In addition, transcriptional repressor Kruppel-like factor 3 (Klf3), which is encoded by *klf3*^*zko352*^, can inhibit the expression of ferric-chelate reductase 1b to promote the maturation of erythroid cells in zebrafish embryos (Crossley et al. 1996; Xue et al. 2015). The homozygous *klf3* mutant was able to survive until adulthood, and the maternal-zygotic *klf3* mutant (hereafter MZ-*klf3*) showed mild erythropoiesis defects (Fig. 4A). Quantitative real-time PCR analysis showed that *klf3* expression was significantly decreased in MZ-*klf3* at 24 hpf, whereas the expression of *klf1* was up-regulated (Fig. 4B). These data indicate that the inconsistent phenotype between *klf3* mutants and morphants could be explained by genetic compensation from other members of the Klf family in the *klf3* mutants.

## Discussion

In this study, we performed large-scale knockout of 1333 genes on zebrafish Chromosome 1 and successfully mutated 1029 of them. Among them, 962 are coding genes and 67 are noncoding genes. Mutations for 438 genes are first reported in this study. We also characterized the features of target sequences and revealed correlation of GC content and nucleotide motifs with the successful mutation rate (success rate) of the CRISPR/Cas9 system. At the molecular level, we reveal that the efficiency of the CRISPR/Cas9 system is highly dependent on the local sequence feature of gRNA target sites. We noticed that 304 genes have not been successfully mutated in our study, which is possibly due to the fact that they are noncoding genes or genes that are close to the telomere. In total, we generated 1039 germline-transmissible alleles corresponding to 636 genes (Supplemental Table S2), of which the information on 693 alleles corresponding to 491 genes has been shared with the ZFIN database (Supplemental Table S2). As the first step, we identified the early morphological defects in 47 of 701 alleles; the detectable phenotype rate is 6.7%, such as in the *plrg1* mutant and in later developmental or metabolic phenotypes in a large number of mutants, such as *runx1*^*zko52a*^ and *gyg2*^*zko624a*^. We found that some mutants exhibit phenotypes inconsistent with their corresponding morphants. More importantly, we discovered that mutants of nearly one in four genes are related to human diseases.

Although there exist some differences, the overall tendencies of the sequence or mutagenesis features of the CRISPR/Cas9 target sites are largely comparable with previous publications based on zebrafish large-scale data sets. (1) Gagnon et al. observed a clear positive correlation between GC content and indel frequency in founder embryos (Gagnon et al. 2014), though Varshney et al. did not detect a significant influence of GC percentage on the mutation rate by examining the germline transmission of the mutations in founder fish (Varshney et al. 2015). We observed a strong positive correlation of the success rate of targeting with the GC content of the 12-nt seed sequences in our data set (Fig. 1), compatible with the result reported by Gagnon et al. (Gagnon et al. 2014). Furthermore, the positive influence of G/C nucleotides on targeting efficiencies was also supported by the observations reported by Moreno-Mateos et al., where nucleotides in the target sites were dominated by G or C enrichment, whereas T and A nucleotides were overall depleted (Moreno-Mateos et al. 2015). (2) We observed that the distribution of PAM sequences did not show significant differences between the positive and negative target sites, although GGG and CGG appear slightly more frequently in the positive sites than in the negative ones (Supplemental Fig. S2A), well reconciling the observation by Moreno-Mateos et al., where G and C were enriched at the first nucleotide of the PAM sequence (Moreno-Mateos et al. 2015). (3) Regarding the nucleotide at position −1 adjacent to (upstream of) the PAM sequence, our result showed that G is relatively enriched in the positive sites, while C is largely enriched in the negative sites (Supplemental Fig. S3C), which is consistent with the observations reported by Moreno-Mateos et al., where there is a strong enrichment for G but depletion in C at this position (Moreno-Mateos et al. 2015). This is also consistent with the observation reported by Gagnon et al., showing that target sites bearing a G adjacent to the PAM motif displayed significantly higher indel frequencies than other bases (Gagnon et al. 2014).

The most valuable resource obtained from this systematic gene perturbation project is the large number of disease-mimicked mutant phenotypes. For example, a recent study reveals that the deficiency of a cilia-related gene, *pkd2*^*zko977a*^, in zebrafish embryos results in phenotypes similar to those of human idiopathic scoliosis (Zhang et al. 2018). Given that it is convenient to perform high-throughput drug screening and genetic modification for disease modeling in zebrafish (Langenau and Zon 2005; Jing and Zon 2011), the abundant disease models of model organisms provide a powerful platform for preclinical trials of drug development (Zon and Peterson 2005; MacRae and Peterson 2015; Leung and Mourrain 2016).

Molecularly, it is essential to further decipher the mechanisms underlying the inconsistency in phenotypes between mutants and morphants. Our finding in *klf3* mutants and morphants demonstrated that the compensatory up-regulation of *klf1* is largely responsible for the phenotypic recovery in *klf3* mutants. Genetic compensation (also known as transcriptional adaptation) is a common physiological phenomenon employed by organisms to accommodate genetic mutations mechanistically through DNA damage response and mutant RNA response (El-Brolosy and Stainier 2017; El-Brolosy et al. 2019; Ma et al. 2019). Additionally, it should be pointed out that maternal effects and MO off-target effects could also contribute to the phenotypic inconsistency (Kok et al. 2015; El-Brolosy and Stainier 2017).

Taken together, the results of our study provide valuable resources for mutant phenotype identification and disease model studies in zebrafish and provide insights into the general working mechanism of the CRISPR/Cas9 system. These findings can facilitate future gRNA design in developmental biology studies and model animal-based drug discovery.

## Methods

### Zebrafish strains

The zebrafish strain Tuebingen was raised in automated facilities at 28.5°C. One-cell stage embryos were used for micro-injection of Cas9 mRNA and gRNA. This study was approved by the Ethical Review Committees from the 24 institutions.

### In vitro synthesis of capped Cas9 mRNA and gRNA, and microinjection

Sequences of humanized Cas9 and zebrafish-codon-optimized Cas9 were cloned into pXT7 vectors separately (Chang et al. 2013; Liu et al. 2014). Capped Cas9 mRNA was synthesized using the mMESSAGE mMACHINE mRNA transcription synthesis kit (Invitrogen, cat.# AM1344). Then, Cas9 mRNA was purified using an RNAclean kit (TIANGEN, cat.# DP412).

Gene-specific gRNAs were designed using the websites (http://zifit.partners.org/ZiFiT/ and https://www.benchling.com/crispr/). gRNAs were synthesized in vitro with a PCR product-based approach, as previously reported (Chang et al. 2013). All tested gRNAs are listed in Supplemental Table S1.

Two hundred picograms of humanized Cas9 or zebrafish-codon-optimized Cas9 mRNA and 50–100 pg of gRNA were co-injected into zebrafish embryos at the one-cell stage.

### Generation and identification of mutants, and phenotype observation

Direct embryo injection with gRNAs and Cas9 mRNA generates the F_0_ line with mosaic mutation, which usually cannot result in embryonic lethality. Further outcross of the F_0_ line with the wild-type line was performed to generate F_1_ heterozygous lines. Finally, the F_2_ mutant line with homozygous mutation was obtained via incross of F_1_ heterozygotes.

F_0_ embryos were identified by Sanger sequencing, T7E1 assay, and restriction enzyme digestion, and gRNA efficiencies were evaluated according to previous descriptions (Chang et al. 2013; Liu et al. 2014). Detailed information for heritable germline transmission strains can be found in Supplemental Table S2. The F_1_ or F_2_ mutant lines were genotyped through Sanger sequencing of PCR fragments covering the gRNA target sites. All the frame-shifted mutants were collected by the China Zebrafish Resource Center, and the genotype of each allele was verified by Sanger sequencing.

The male and female of the F_1_ alleles with the same genotype were crossed to obtain F_2_ progeny. The F_2_ embryos were raised in 0.3× Danieau Buffer at 28.5°C and regularly observed under stereomicroscope for morphological defects before 10 d postfertilization.

### Whole-mount in situ hybridization

Whole-mount in situ hybridization was performed as previously described (Wang et al. 2011). The Digoxigenin-labeled RNA probes detecting *plrg1*, *gsc*, *ntl, sox17*, *lmo2*, *gata1*, *scl*, *hbbe2*, *hbbe1*, *cmyb*, and *rag1* were transcribed using T7 or SP6 polymerase.

### MO microinjection and mRNA overexpression

The antisense morpholinos including *plrg1* MO and *tp53* MO were purchased from GeneTools. The detail sequences are shown as follows: *plrg1* MO (5′-TGCTTCTGCACGTCCTCGGTCATGT-3′), *tp53* MO (5′- GCGCCATTGCTTTGCAAGAATTG-3′). For injection into zebrafish embryos at the one- to four-cell stage, 0.16 ng *plrg1* MO and 4 ng *tp53* MO were used.

*plrg1* full-length mRNA was synthesized from zebrafish cDNA using an mMESSAGE mMACHINE mRNA transcription synthesis kit (Invitrogen, cat.# AM1344). Then, *plrg1* mRNA was purified using an RNAclean kit (TIANGEN, cat.# DP412). Seventy-five picograms of *plrg1* mRNA was used for injection into zebrafish embryos at the one-cell stage.

### Quantitative real-time PCR

Total RNAs from the whole embryos of siblings and mutants were extracted using TRIzol reagent (Life Technologies, cat.# 15596018). The cDNA was reversely transcribed from the total RNAs using M-MLV Reverse Transcriptase (Promega, cat.# M1701). Then, the cDNA was used as a template for quantitative real-time PCR.

### TUNEL assay

A TUNEL assay was performed using an In Situ Death Detection kit, TMR red (Roche, cat.# 12156792910) following the manufacturer's instructions. Briefly, *plrg1* siblings and mutants at 48 h postfertilization were fixed with 4% paraformaldehyde and permeabilized with Proteinase K (10 μg/mL) for 20 min. Subsequently, the embryos were incubated with the TUNEL reaction mixture at 4°C. After the reaction, confocal microscopy was performed using a Nikon confocal A1 laser microscope (Nikon).

## Data access

All the data related to available alleles from this study have been submitted to the China Zebrafish Resource Center (CZRC) at the following link: http://www.zfish.cn/TargetList.aspx, and to the Zebrafish Information Network (ZFIN) at the following link: http://zfin.org/action/publication/ZDB-PUB-171002-4/feature-list.

## ZAKOC Consortium

Yonghua Sun,^26^ Luyuan Pan,^26^ Houpeng Wang,^26^ Weixun Xie,^26^ Mudan He,^26^ Ding Ye,^26^ Kuoyu Li,^26^ Feng Xiong,^26^ Liyue Liu,^26^ Linglu Li,^26^ Yun Zhang,^26^ Bo Zhang,^27^ Da Liu,^27^ Zhenchao Cheng,^27^ Yingying Hu,^27^ Qian Wu,^27^ Zhou Luo,^27^ Yutian Zhang,^27^ Yingdan Wu,^27^ Wenyuan Li,^27^ Lingfei Luo,^28^ Jianlong Ma,^28^ Dashuang Mo,^28^ Pengcheng Cai,^28^ Jinzi Chen,^28^ Junhui Sun,^28^ Yang Zhou,^28^ Chuan Wu,^28^ Rui Ni,^28^ De-Li Shi,^29^,^54^ Ming Shao,^29^ Yan-Jun Zhang,^29^ Li-Jun Feng,^29^ Xiao-Ning Cheng,^29^,^54^ Ji-Tong Li,^29^ Yan-Yi Xing,^29^ Yan Zhang,^29^ Ya-Ping Meng,^29^ Bei-Bei Yu,^29^ Han Wang,^30^ Jian Huang,^30^ Shuqing Zhang,^30^ Cheng Ji,^30^ Yicheng Tan,^30^ Jingjing Wang,^30^ Fanmiao Zhang,^30^ Mingyong Wang,^30^ Guodong Huang,^30^ Zhaomin Zhong,^30^ Wei Zhang,^30^ Zongbin Cui,^26^ Yong Long,^26^ Guili Song,^26^ Xiaohui Li,^26^ Xixi Li,^26^ Kai Chen,^26^ Jing Ren,^26^ Junjun Yan,^26^ Qing Li,^26^ Honghui Huang,^28^ Hua Ruan,^28^ Shicheng Zhu,^28^ Xuejiao Chen,^28^ Jingjing Pan,^28^ Faming Jiang,^28^ Jiehui Chen,^28^ Chao Huang,^28^ Guoping Dong,^28^ Xiaogui Yi,^28^ Ying Cao,^31^ Ruikun Hu,^31^ Weilai Huang,^31^ Jiangfang Liu,^31^ Hong WangXiao,^31^ Dong Shu,^32^ Jianhong Xia,^32^ Shaoyang Zhao,^32^ Pengtao Wang,^32^ Xiuhua Wu,^32^ Wenqing Zhang,^33^ Yiyue Zhang,^33^ Mengchang Xu,^33^ Wei Liu,^33^ Zhibing Huang,^33^ Qing Lin,^33^ Mei Wu,^33^ Jianfeng Zhou,^34^ Aibo Sheng,^34^ Peipei Tang,^34^ Xiaoxia Gong,^34^ Wei Mou,^34^ Congcong Zu,^34^ Yun Li,^34^ Ling Lu,^34^ Yunzhang Liu,^34^ Xiaozhi Rong,^34^ Jianyang Chen,^34^ Jiulin Du,^35^ Jiwen Bu,^35^ Xufei Du,^35^ Tingting Liu,^35^ Shanye Gu,^35^ Qingshun Zhao,^36^ Zhangji Dong,^36^ Xiaohua Dong,^36^ Shasha Cao,^36^ Yunyun Yue,^36^ Chun Gu,^36^ Meijing Liu,^36^ Jun Chen,^37^ Hongjian Gong,^37^ Hanbing Zhong,^38^,^39^ Xuanjun Yang,^38^,^39^ Jiahao Chen,^38^ Qiushi Xu,^38^ Tao P. Zhong,^40^ Daqin Jin,^40^ Peilu She,^40^ Jianjian Sun,^40^ Kaa Seng Lai,^40^ Li Li,^28^ Yu Zhang,^28^ Yanyan Shi,^28^ Fangying Zhao,^28^ Jing-Wei Xiong,^41^ Meijun Pang,^41^ Lu Gao,^41^ Lei Lei,^41^ Jinrong Peng,^42^ Jane Lo,^42^ Wuhan Xiao,^26^ Xiaolian Cai,^26^ Gang Ouyang,^26^ Jian Zhang,^43^,^44^ Weirui Ma,^45^ Liyun Miao,^45^ Jihua Yao,^46^ Yu Hu,^46^ Zhan Yin,^26^ Xianming Mo,^47^ Xue Li,^47^ Gang Peng,^48^ Cuizhen Zhang,^48^ Fenghua Wang,^48^ Jun Zhu,^49^ Ruimeng Yang,^49^ Hao Yuan,^49^ Yan Chen,^50^ Yi Pan,^50^ Yong Zhou,^51^ Dong Liu,^38^ Feng Zhao,^38^ Weijun Pan,^50^ Feng Liu,^52^ Yanyan Ding,^52^ Yuanyuan Xue,^52^ Lu Wang,^52^ Yifan Zhang,^52^ Dongyuan Ma,^52^ Xinyan Lu,^52^ Shuai Gao,^52^ Jun Xia,^52^ Zuoyan Zhu,^26^ Anming Meng,^53^ Xingfeng Liu,^53^ Juhui Qiu,^53^ Bo Gong,^53^ Luxi Chen,^53^ Cong Xiong,^53^ Likun Yao,^53^ Cencan Xing,^53^ Yixia Wang,^53^ Weimin Shen,^53^ Jiawei Sun,^53^

## Supplementary Material

Supplemental Material

## Footnotes

26 State Key Laboratory of Freshwater Ecology and Biotechnology, Institute of Hydrobiology, Innovation Academy of Seed Design, Chinese Academy of Sciences, Wuhan, Hubei, 430072, China

27 Key Laboratory of Cell Proliferation and Differentiation of the Ministry of Education, Peking University Genome Editing Research Center, College of Life Sciences, Peking University, Beijing, 100871, China

28 School of Life Sciences, Southwest University, Chongqing, 400715, China

29 School of Life Sciences, Shandong University, Qingdao, 266237, China

30 Center for Circadian Clocks, Soochow University, Suzhou, Jiangsu, 215123, China

31 School of Life Sciences and Technology, Tongji University, Shanghai, 200092, China

32 Guangzhou Institutes of Biomedicine and Health, Chinese Academy of Sciences, Guangzhou, Guangdong, 510530, China

33 Division of Cell, Developmental and Integrative Biology, School of Medicine, South China University of Technology, Guangzhou, Guangdong, 510006, China

34 School of Medicine and Pharmacy, Ocean University of China, Qingdao, Shandong, 266100, China

35 State Key Laboratory of Neuroscience, Institute of Neuroscience, Chinese Academy of Sciences, Shanghai, 200031, China

36 Model Animal Research Center, Nanjing University, Nanjing, Jiangsu, 210093, China

37 College of Life Sciences, Zhejiang University, Hangzhou, Zhejiang, 310058, China

38 Department of Biology, South University of Science and Technology of China, Shenzhen, Guangdong, 518055, China

39 Institute of Chinese Medical Sciences, University of Macau, Macau, China

40 Institute of Biomedical Sciences, Shanghai Key Laboratory of Regulatory Biology, East China Normal University, Shanghai, 200062, China

41 College of Life Sciences, Institute of Molecular Medicine, Peking University, Beijing, 100871, China

42 College of Animal Sciences, Zhejiang University, Hangzhou, Zhejiang, 310058, China

43 State Key Laboratory for Conservation and Utilization of Bio-Resources, Kunming, 650091, China

44 Center for Life Sciences, School of Life Sciences, Yunnan University, Kunming, 650091, China

45 State Key Laboratory of Molecular Developmental Biology, Institute of Genetics and Developmental Biology, Chinese Academy of Sciences, Beijing, 100101, China

46 School of Life Sciences, Fudan University, Shanghai, 200433, China

47 State Key Laboratory of Biotherapy, West China Hospital, Sichuan University, Chengdu, Sichuan, 610041, China

48 Institutes of Brain Science, Fudan University, Shanghai, 200433, China

49 Sino-French Research Center for Life Sciences and Genomics, Rui-Jin Hospital, Shanghai Jiao Tong University School of Medicine, Shanghai, 200025, China

50 Institute of Health Sciences, Chinese Academy of Sciences & Shanghai Jiao Tong University School of Medicine, Shanghai, 200025, China

51 CAS Key Laboratory of Nutrition, Metabolism and Food Safety, Shanghai Institute of Nutrition and Health, Chinese Academy of Sciences, Shanghai, 200031, China

52 State Key Laboratory of Membrane Biology, Institute of Zoology, Chinese Academy of Sciences, Beijing, 100101, China

53 School of Life Sciences, Tsinghua University, Beijing, 100084, China
