# Supplementary material for: Systematic genome editing of the genes on zebrafish Chromosome 1 by CRISPR/Cas9
Source: Genome Res. 2020 Jan;30(1):118–26. doi: 10.1101/gr.248559.119 (PMC6961580; doi:10.1101/gr.248559.119)
Supplement: Supplemental Material [file supp_30_1_118__index.html]

Systematic genome editing of the genes on zebrafish Chromosome 1 by CRISPR/Cas9 — Supplemental Material 

# Systematic genome editing of the genes on zebrafish Chromosome 1 by CRISPR/Cas9

## Supplemental Material

- Supplemental\_Figures.docx
- Supplemental\_Table\_S1.R3.xlsx
- Supplemental\_Table\_S2-R3.xlsx
- Supplemental\_Table\_S3\_R3.xlsx
- Supplemental\_Data\_file\_S1\_R3.zip
