# Supplementary material for: Systematic genome editing of the genes on zebrafish Chromosome 1 by CRISPR/Cas9
Source: Genome Res. 2020 Jan;30(1):118–26. doi: 10.1101/gr.248559.119 (PMC6961580; doi:10.1101/gr.248559.119)
Supplement: Supplemental Material [file supp_gr.248559.119_Supplemental_Figures.docx]

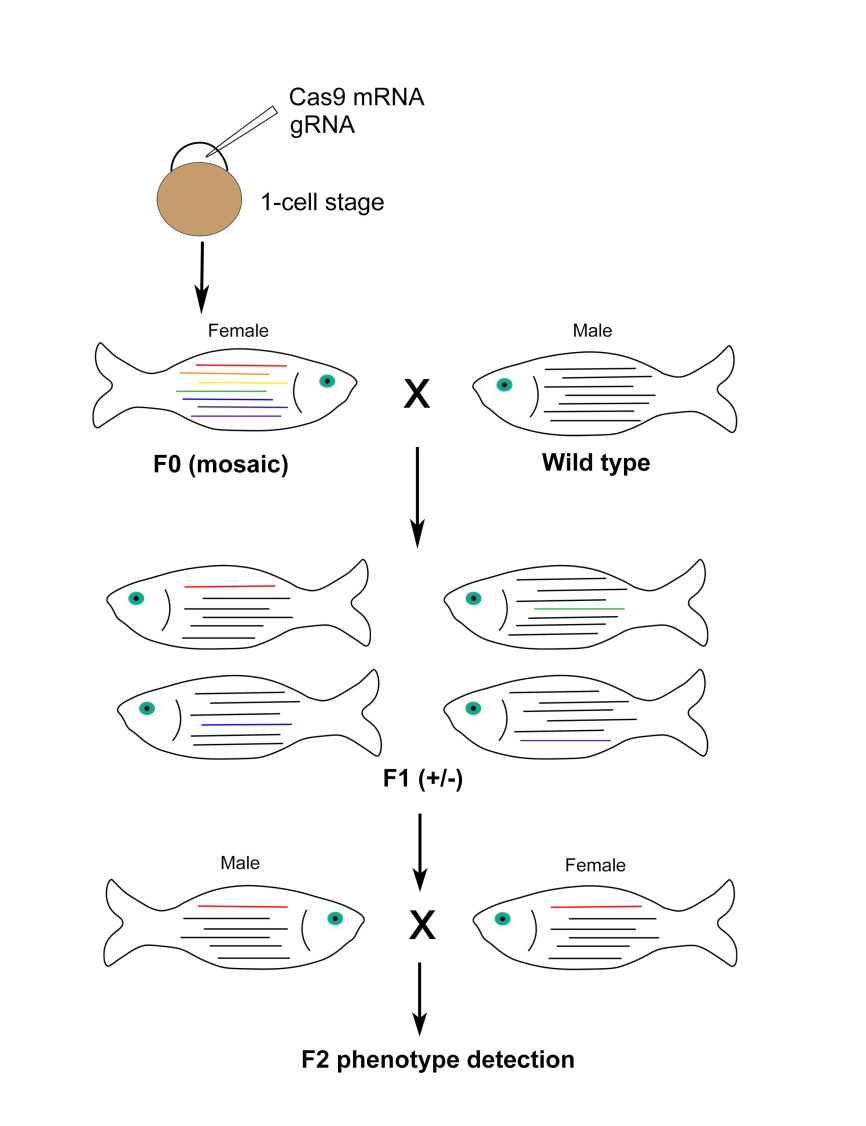


**Supplemental Figure 1:** Overview of mutagenesis using CRISPR/Cas9 and phenotype detection. Cas9 mRNA and gRNA were co-injected into zebrafish embryos at 1-cell stage. F_0_ embryos were identified by Sanger sequencing, T7E1 assay and restriction enzyme digestion. Then, the F_0_ fishes were outcrossed with wild type to acquire F_1_ (heterozygous) mutants identified by Sanger sequencing. The F_1_ mutants were crossed and F_2_ embryos were collected for phenotype detection.

**
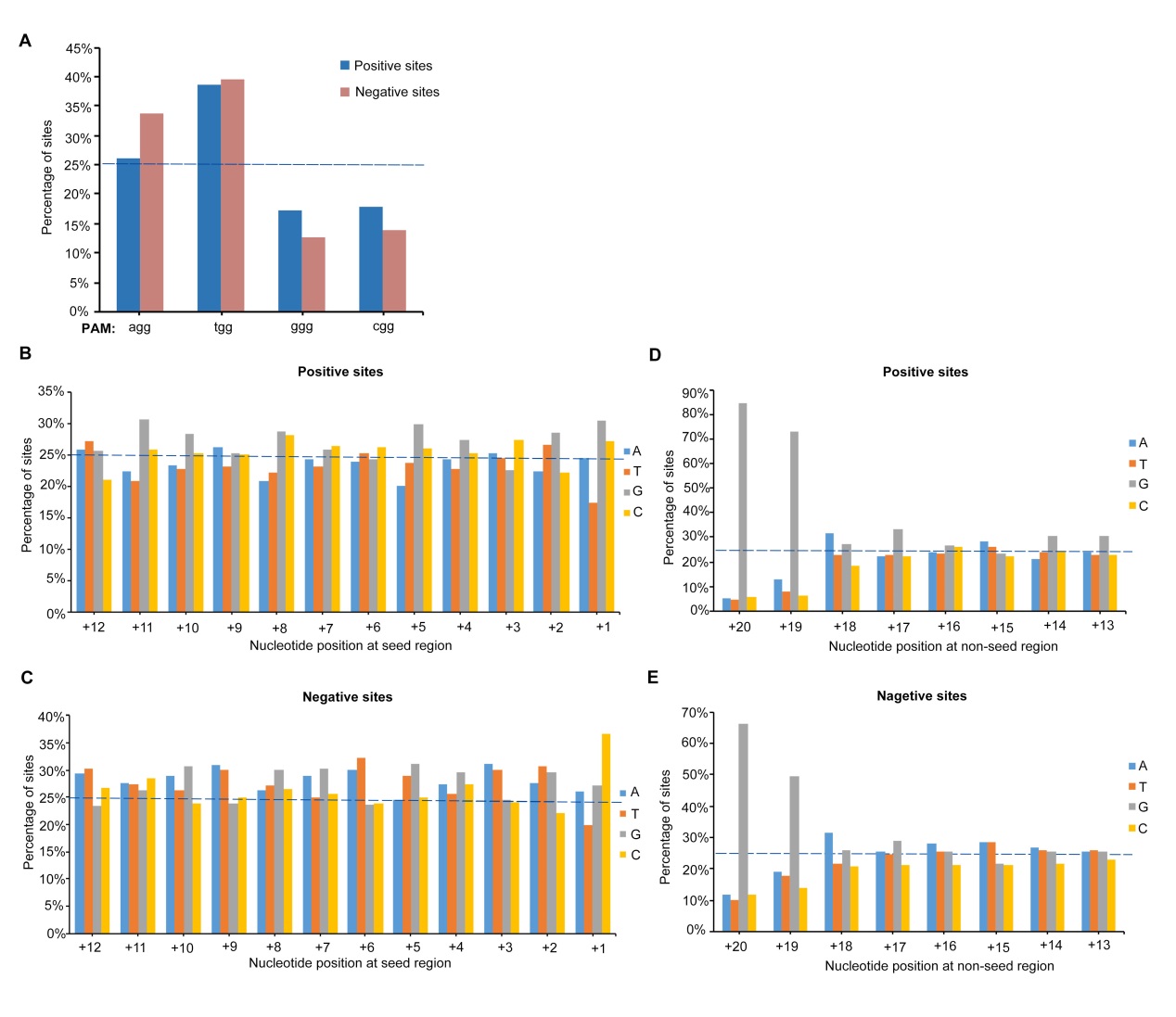
**

**Supplemental Figure 2:** Nucleotide distribution at each position of all the tested target sites**.** (*A*) The distribution of protospacer adjacent motif (PAM) sequences in all the tested target sites. (*B*) Single nucleotide distribution at each position of the 12-nt seed region in the 1,086 positive target sites. The number in the X-axis indicates the relative position of the corresponding nucleotides to the PAM sequence, with +1 being the first nucleotide upstream to the PAM. (*C*) Single nucleotide distribution at each position of the 12-nt seed region in the 1,191 negative target sites. The number in the X-axis indicates the relative position of the corresponding nucleotides to the PAM sequence, with +1 being the first nucleotide upstream to the PAM. (*D*) Single nucleotide distribution at each position of the 8-nt non-seed region (positions +13~+20) in the 1,086 positive target sites. The significantly high percentage of base G at the last two positions (+19 and +20) is due to the obligate requirement for the presence of two terminal Gs for efficient in vitro transcription of gRNAs by T7 RNA polymerase. (*E*) Single nucleotide distribution at each position of the 8-nt non-seed region (positions +13~+20) in the 1,191 negative target sites. The significantly high percentage of base G at the last two positions (+19 and +20) is due to the obligate requirement for the presence of two terminal Gs for efficient in vitro transcription of gRNAs by T7 RNA polymerase.


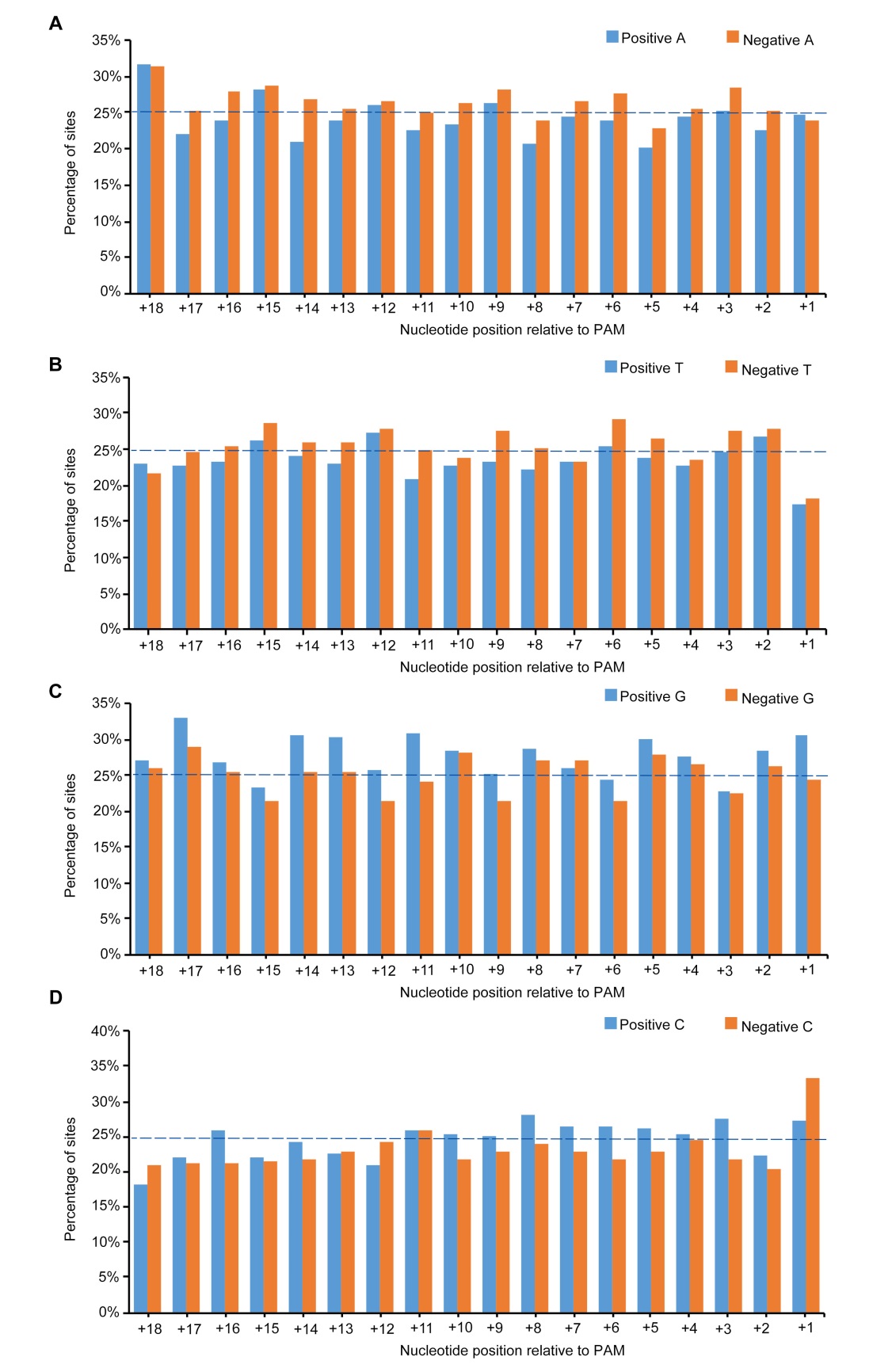


**Supplemental Figure 3:** Comparison of single nucleotide composition between positive sites and negative sites at each position of the 18-nt target sequences adjacent to PAM**.** (*A*) Distribution of nucleotide A (among the four nucleotides) at each position of the 18-nt adjacent sequence in both positive and negative target sites. The number in the X-axis indicates the relative position of base A to the PAM sequence, with +1 being the first nucleotide upstream to the PAM. (*B*) Distribution of nucleotide T (among the four nucleotides) at each position of the 18-nt adjacent sequence in both positive and negative target sites. The number in the X-axis indicates the relative position of base T to the PAM sequence, with +1 being the first nucleotide upstream to the PAM. (*C*) Distribution of nucleotide G (among the four nucleotides) at each position of the 18-nt adjacent sequence in both positive and negative target sites. The number in the X-axis indicates the relative position of base G to the PAM sequence, with +1 being the first nucleotide upstream to the PAM. (*D*) Distribution of nucleotide C (among the four nucleotides) at each position of the 18-nt adjacent sequence in both positive and negative target sites. The number in the X-axis indicates the relative position of base C to the PAM sequence, with +1 being the first nucleotide upstream to the PAM.


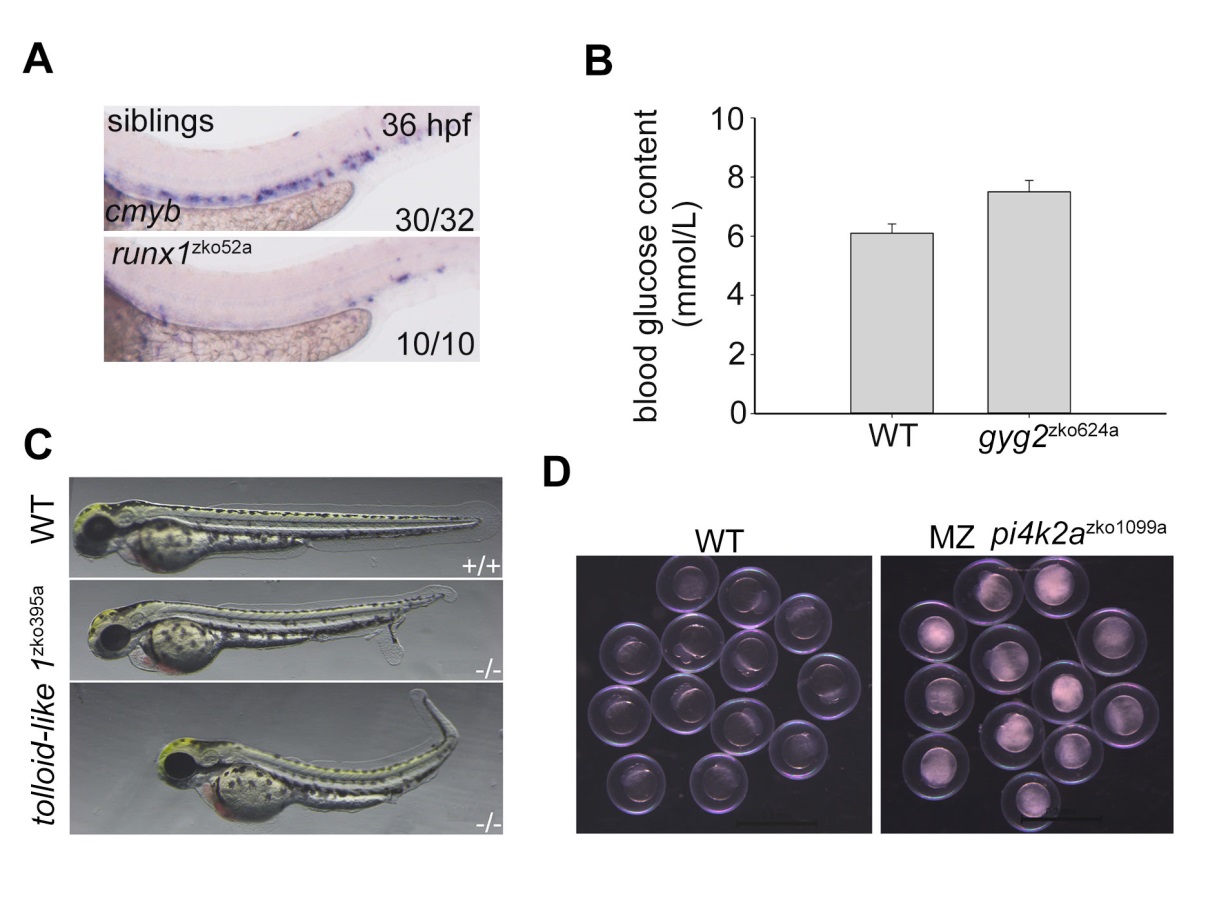


**Supplemental Figure 4:** Characterization of development and disease-related phenotypes in zebrafish zko mutants. (*A*) Whole mount in situ hybridization (WISH) showing the expression of *cmyb* at 36 hours post fertilization (hpf) in siblings (left panel) and *runx1* mutants (right panel). The *cmyb* expression in aorta-gonad-mesonephros at 36 hpf was decreased in *runx1* mutants compared to siblings. (*B*) Blood glucose content is increased in *gyg2* heterozygotes compared to wild-type. (*C*) Microscopy imaging showing that *tolloid-like 1* mutant lacks ventral fin. (*D*) Microscopy imaging showing that the embryos of maternal-zygotic (MZ) *pi4k2a* mutants are not transparent.

**
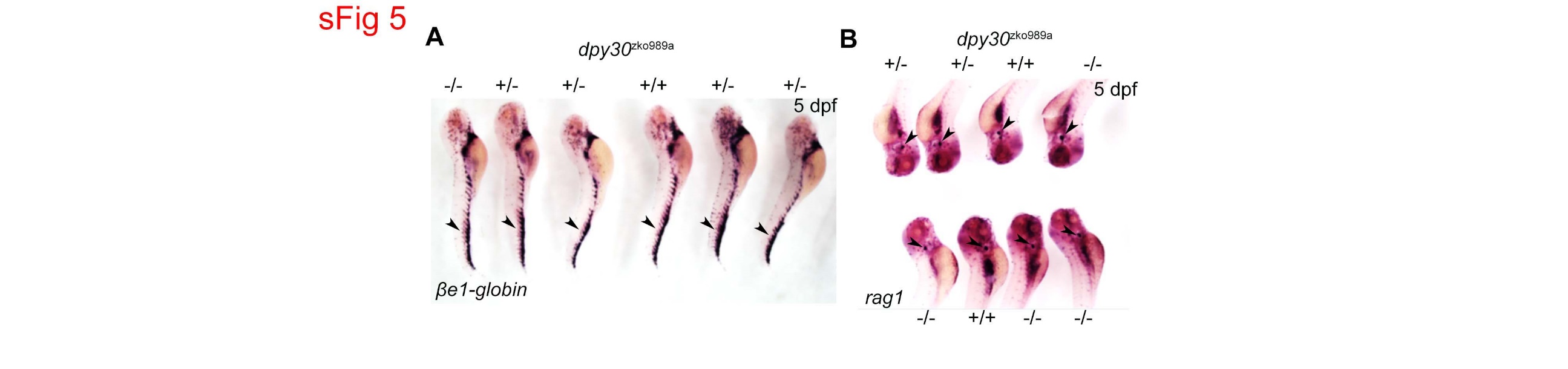
**

**Supplemental Figure 5:** Characterization of erythropoiesis and lymphopoiesis phenotypes in *dpy30* mutants. (*A*) WISH showing the expression of *βe1*-*globin* at 5 days post fertilization (dpf) in *dpy30* siblings and mutants. (*B*) WISH showing the expression of *rag1* at 5 dpf in *dpy30* siblings and mutants. The black arrowheads indicate the expressions of *βe1*-*globin* in erythroid lineage and *rag1* in T lymphoid lineage, respectively.
